# Should Schools Expect Poor Physical and Mental Health, Social Adjustment, and Participation Outcomes in Students with Disability?

**DOI:** 10.1371/journal.pone.0126630

**Published:** 2015-05-12

**Authors:** Sharmila Vaz, Reinie Cordier, Marita Falkmer, Marina Ciccarelli, Richard Parsons, Tomomi McAuliffe, Torbjorn Falkmer

**Affiliations:** 1 School of Occupational Therapy and Social Work, Curtin University, Perth, Western Australia, Australia; 2 School of Education and Communication, CHILD programme, Institution of Disability Research Jönköping University, Jönköping, Sweden; 3 School of Pharmacy, Curtin University, Perth, Western Australia, Australia; 4 James Cook University, College of Healthcare Sciences, Occupational Therapy, Townsville, Queensland, Australia; 5 Rehabilitation Medicine, Department of Medicine and Health Sciences (IMH), Faculty of Health Sciences, Linköping University & Pain and Rehabilitation Centre, UHL, County Council, Linköping, Sweden; University of Perugia, ITALY

## Abstract

The literature on whether students with disabilities have worse physical and mental health, social adjustment, and participation outcomes when compared to their peers without disabilities is largely inconclusive. While the majority of case control studies showed significantly worse outcomes for students with disabilities; the proportion of variance accounted for is rarely reported. The current study used a population cross-sectional approach to determine the classification ability of commonly used screening and outcome measures in determining the disability status. Furthermore, the study aimed to identify the variables, if any, that best predicted the presence of disability. Results of univariate discriminant function analyses suggest that across the board, the sensitivity of the outcome/screening tools to correctly identify students with a disability was 31.9% higher than the related Positive Predictive Value (PPV). The lower PPV and Positive Likelihood Ratio (LR^+^) scores suggest that the included measures had limited discriminant ability (17.6% to 40.3%) in accurately identifying students *at-risk* for further assessment. Results of multivariate analyses suggested that poor health and hyperactivity increased the odds of having a disability about two to three times, while poor close perceived friendship and academic competences predicted disability with roughly the same magnitude. Overall, the findings of the current study highlight the need for researchers and clinicians to familiarize themselves with the psychometric properties of measures, and be cautious in matching the function of the measures with their research and clinical needs.

## Introduction

Supporting the inclusion and participation of all students in the school setting is emphasised as a universal need [1,2]. The concept of inclusion is based on a notion of social justice that advocates equal access to all educational opportunities for all students, regardless of the presence of a disability or any form of disadvantage [3]. Educational policies in developed countries have responded to this social justice agenda in different ways. In Australia, students with disabilities continue to experience barriers to equitable participation [4], despite the government’s commitment to inclusive education reported in an array of documents and policies [1,2,5]. According to the 2009 Australia national records, 65.9% of 5–20 year old students with disabilities attended mainstream schools; 24.3% attended special classes within mainstream schools, and 9.9% attended special education schools. This pattern was consistent regardless of the severity of the disability [6].

Physical placement per se of students with disabilities in a mainstream setting does not automatically result in the school being perceived as inclusive by the student [7,8]. Instead, students with disabilities continue to experience barriers to equitable participation, some due to the prejudices held by the general population; including beliefs about their needs, rights, vulnerabilities and competencies [9]. Research findings to date suggest that a person’s diagnostic category does not affect the intensity and diversity of his or her participation [10,11].

Most studies of statistical associations between child characteristics, type of disability, and participation outcomes, report weak to moderate correlations [12–14]. One possible explanation for these moderate to weak associations is that disability is only one of several factors that affect participation and that the effects of other factors are stronger. Important factors for predicting participation in school activities of pupils with disabilities are child characteristics, such as autonomy, locus of control and engagement; environmental factors such as adaptations of the environment; and the attitudes of teachers and peers [10–14]. Students with disabilities are reported to participate less in structured and unstructured activities, and to experience limited classmate interaction and recess participation compared with their peers without disabilities [15–18]. These reports of systemic exclusion prompted the need for the current study, in which physical health, mental health (self-concept, coping), social adaptation (social skills, school belongingness, loneliness and social dissatisfaction) and participation in school activities are investigated in relation to having a disability. Each of these outcomes are defined and described in relation to students with disabilities.

### Physical and mental health functioning

#### Physical health

Population based studies report that having a disability has a significant impact on children’s health and educational functioning [19]. The extent of this impact appears to be much greater among children with multiple disabilities. In fact, the impact on school performance has been found to be even more pronounced for children reported to have learning disabilities in addition to their physical impairments [19].

#### Mental health

Contemporary research indicates that conceptualising mental health as a unidimensional construct is limiting [20]. Mental health is a state of emotional and social wellbeing that allows the individual to realise his or her own abilities, cope with normal stresses of life, undertake productive activities, experience meaningful personal relationships, and make a meaningful contribution to his or her community [21,22]. Mental health should arguably be seen to reflect a multi-faceted and interactive construct encompassing “the absence of dysfunction in psychological, emotional, behavioural and social spheres” and “optimal function or well-being” (p. 128) [23]; not just the absence of disease.

Having a disability, irrespective of type, increases the risk of developing mental health problems and disorders because of associated adverse individual and environmental factors [24–26]. Estimates suggest that young people with an intellectual disability manifest behaviours and experiences that may be indicative of mental health problems, three to four times more often than their peers without disability; with between 4% and 18% having a mental health diagnosis [27]. Mental health problems in young people with a disability are often undiagnosed and untreated; and impact on them acquiring skills necessary for their successful integration into the community [27].

#### Self-concept

Self-concept refers to an individual’s belief about his or her behavioural capabilities in a range of skills, knowledge and attitudes, that are drawn from various cognitive, motor and social skills [28,29]. These beliefs are reflections of the person’s actual abilities and internalisation of the feedback obtained from significant others [30], and social comparison with others in the same setting [31]. A person’s self-concept undergoes varying degrees of adaptation during different life stages and experiences [32]. Different social environments are likely to influence an individual's self-concept in different ways.

Literature on the impact of disability on the self-concept of students in mainstream educational settings is inconclusive, with inconsistencies reported between studies dependent on: a school’s mainstreaming philosophy; the dimension of self-concept explored; and nature and severity of the participants’ disability. For example, students with learning disabilities reportedly have lower academic self-concept when compared with their typically developing peers [33]; but findings with regards to their global self-esteem are mixed, with some studies suggesting lower global self-esteem in the learning disability subgroup [34], and others reporting no group differences [35,36]. Students with hearing, learning, and physical disabilities are also reported to have lower social and academic competence when compared to their non-disabled peers, but show no differences in the level of reported physical self-concept [33,37]. The phenomenon of elevated self-concept among students with externalising behaviors is also widely reported in the disability literature [38], and is hypothesised to serve as a protective factor, buffering the person from the negative effects of social and academic failures [39].

#### Coping skills

Coping involves the use of cognitive or behavioural strategies to manage stress [40], and are related to self-regulation; a core component of healthy adaptation [41]. Perceived competence and coping skills may reduce psychological distress and buffer the deleterious effects of stress, resulting in better adjustment [42,43]. However, few studies to date have reviewed the coping skills of students with disabilities in an educational context. Existing work in the area suggests that students with learning disabilities use passive cognitive avoidance and more wishful thinking coping strategies when faced with academic stress-related events [44,45]. They also tend to receive less peer support when coping with academic or interpersonal problems, when compared with students without disability [46].

### Social adjustment

#### Social skills

Social skills include socially acceptable learned behaviours that enable individuals to interact successfully with others and avoid socially undesirable responses [47]. This definition of social skills is a hybrid of the peer acceptance and behavioural definitions, and is the most socially valid in the sense of predicting important social outcomes for children [47]. Development of social skills is regarded as a fundamental task for all children [48]. Acquisition or performance deficits in social skills may impede the quality of an individual’s social relationships and social adjustment [49–51]. For example, deficits have been linked to social adjustment problems, such as peer rejection, loneliness, reduced school belongingness, and early withdrawal from school. A variety of pejorative outcomes beyond the school setting including substance abuse, chaotic personal lives, and limited or absent post-secondary educational experiences, have also been reported among students with disabilities who have social skills deficits [52]. Given the difficulties and the associated risk of poor social development, it is imperative for educators and health professionals to identify and provide interventions for children who experience problems in this developmental area [51].

#### Belongingness in school

The feeling of belongingness represents an active internal experience of a strong psychological connection [53,54]. School belongingness is defined in terms of the degree to which a student feels accepted and included within the school [55]. When students have a sense of belonging in school, they believe that the school community is incomplete without them, and vice versa. Severity of disability has been shown to influence students’ perceptions of belongingness in school. For example, research suggests that students with mild learning disabilities have levels of school belongingness similar to their typically developing peers, despite having lower academic performance and behavioural vulnerability [56]. For students with moderate and severe disabilities, school belongingness appeared to be dependent on the students’ relationships within classroom-based social groups [57] and their involvement in classroom activities [58].

#### Loneliness and social dissatisfaction in school

There are several definitions of loneliness in research literature. Some scholars consider it to be a unidimensional construct that is a discrepancy between desired and obtained social contacts [59]. Other researchers consider loneliness to be a multi-dimensional entity, comprised of several individual and relational aspects [60]. It is widely believed that school-aged children have a complex and multi-dimensional conceptualisation of loneliness [61]; however, differences in conceptualisation are inconsistently described in the literature. Indications about one’s social network (i.e., being alone) and reflection on subjective sadness have been specified by 9–11 year old students in an Australian sample [61]. Not all students conceptualised loneliness as a multi-faceted entity. Almost 40% of the children in the Australian sample described loneliness without referencing distressing emotions; 10% described loneliness without referencing social deficits and more than 80% did not conceptualise being alone with loneliness. References to self-attributions (e.g., having no courage to talk about their situation, being in one’s own world, or being different) were used when describing loneliness [61]. These findings demonstrate the highly subjective nature of loneliness, which has been identified as a key reason for the difficulties in understanding how individuals experience loneliness [62].

The literature presents mixed findings about the impact of disability on students’ perceptions of loneliness at school. Some findings suggest students with learning disabilities who are enrolled in mainstream schools are less socially accepted, have fewer friends, and feel more lonely when compared to their peers without disabilities [63–65]. Other studies report no group differences in loneliness between students with physical disability and their typically developing peers; but those with Autism Spectrum Disorders have been found to have twice the loneliness of other disability subgroups [66]. Students with learning and other physical disabilities have a higher degree of social dissatisfaction with their peer relationships [67]. Difficulties in reading and processing social cues, and difficulties in expressing emotions in social situations have been identified as potential contributors to the increased vulnerability and propensity for rejection by peers among students with disability [68,69].

### Summary of the Literature

In summary, the literature on differences in physical and mental health, social adjustment, and participation outcomes in students with and without disabilities is largely inconclusive. The research studies included in this introduction used convenience samples and focussed on a few disability subtypes (mainly mild intellectual disability, learning disability, Attention Deficit Hyperactivity Disorder [ADHD] and Autism Spectrum Disorders). Case-control designs were used to identify differences between students with and without disability, and commonly used models to detect differences included a simple regression model using the *t-test*, or a *Mean-value* difference using the *t-tests* or the *Mann-Witney U-test*. The majority of these studies showed significant between-group differences; however, the proportion of variance accounted for was low. Therefore, to truly establish whether or not students differed on a variety of outcome measures based on disability, a cross-sectional design with a representative sample of students with and without disability, in mainstream schools is needed. Using a population cross-sectional approach, the classification accuracy of several outcome measures used with school children can be estimated.

Consequently, the current study aimed to: a) assess the classification ability of commonly used screening and outcome measures in determining the disability status of primary school children; and b) identify the variables, if any, that best predicted the presence of disability.

## Method

### Participants

Cross-sectional data from 395 students, parents and class-teachers from 75 primary schools and 77 classrooms across metropolitan Perth and other major urban centres of Western Australia were used. Data for this study were drawn from a large longitudinal study on the factors associated with student adjustment across the primary-secondary transition [70,71]. Students were categorised as having a disability if they were reported to have a disability by their primary caregiver, which had an impact on the student’s daily functioning. To be eligible for the study, their parent(s)/care-giver(s) needed to confirm that they were attending a mainstream class for at least 80% of their school hours per week, with support provided as required. Thus, a broad definition was used to categorise students into the disability group. Further details on the study’s design, recruitment, data collection, and sample characteristics have been published elsewhere [70]. Participation in the study was voluntary. Informed written consent was obtained from school principals, parents, teachers, and written assent was obtained from students to participate in this study. All participants were made aware that they could withdraw from the study at any time without justification or prejudice. Ethics approval was obtained from Curtin University Health Research Ethics Committee, in Western Australia (WA) (approval number HR 194/2005).

### Data collection instruments

#### Short Form Health Survey (SF-36)

Items from the SF-36, a multipurpose short form generic measure of health status were used to gain an understanding of parents’ perception of their child’s physical and overall health [72].

#### Strengths and Difficulties Questionnaire

The Strengths and Difficulties Questionnaire (SDQ) [73] was developed as a brief screening tool that describes children and adolescents’ behaviours, emotions and relationships. The SDQ aims to assess both negative and positive attributes of behaviour across five domains (namely conduct problems, emotional symptoms, hyperactivity, peer relationships and prosocial behaviour). The author suggests that the SDQ can be used for screening, as part of a clinical assessment, as a treatment outcome measure, and as a research tool [73,74]. The parent version of the Strengths and Difficulties Questionnaire (SDQ) was used to measure overall mental health functioning [73,75,76]. The overall score was derived by summing students’ emotional, conduct problems, hyperactivity/inattention, and peer relationship scores. Higher scores indicate poorer overall mental health functioning.

The parent version of the SDQ is reported to have moderate to high weighted mean internal consistency (α = 0.53–0.80) [77]. Discriminate and predictive validity of the measure has previously been reported [77]. For the calculation of screening efficiency, the SDQ total and subscale scores were classified into three categories (‘unlikely’, ‘possible/ query’ and ‘probable/ of concern’). On the basis that approximately 10% of the child and adolescent populations exhibit some kind of mental health problem, the ‘probable/ of concern’ range included scores above the 90^th^ percentile. To calculate the values for screening efficiency, the SDQ groups were dichotomised into ‘diagnosis’ and ‘no diagnosis’ [78–80]. This dichotomisation was necessary to calculate the screening efficiency in terms of sensitivity, specificity, positive predictive value (PPV), and negative predictive value (NPV) (S1 Appendix). The SDQ has been found to distinguish between those children/adolescents receiving treatment and those who are not, and between particular diagnoses or problematic behaviour, at least as well as other, more established instruments like the Rutter questionnaires, the Child Behaviour Checklist (CBCL), and the Youth Self-Report (YSR) [73,75,79,81,82]. The SDQ has also been widely used in clinical populations [83] and with adolescents with intellectual disability [84,85].

#### Adolescent Coping Scale

The Adolescent Coping Scale (ACS) is a self-report inventory designed to support young people when examining their own coping behaviour. The ACS helps to measure the usage and helpfulness of coping strategies in general and specific situations [40]. The ACS was designed for use in clinical, educational, and research contexts. This self-report measure is based on the implicit assumption that groups of functional coping actions are more likely to lead to adaptive outcomes, whereas dysfunctional strategies are more likely to result in maladaptive outcomes. The ACS measures what people feel, think, or do to cope [86]. The scale uses a five-point Likert rating system, ranging from 1 (doesn’t apply or don’t do it) to 5 (used a great deal) to rate each item. In line with evidence that suggests that an individual’s choice of coping strategy is mostly consistent [87], the General Form of the instrument that addresses how people cope with concerns in general was used. The short form of the ACS also allows for combining scales to produce measures of three empirically defensible coping styles based on factor analysis. These three coping domains comprise two functional coping styles (i.e., solving the problem, and reference to others), and one dysfunctional coping style (i.e., non-productive coping). Internal consistencies are reported to range from α = 0.50 (reference to others) to 0.66 (non-productive coping) [88]. Test-retest reliabilities for the same subscales on the general form range from *r* = .44 to .84 (Mean *r* = .69) [89].

#### Self-Perception Profile for Adolescents

Items from the Self-Perception Profile for Adolescents (SPPA) measured student perceived competence in domains of academics, athletics, social acceptance, physical appearance, close friendships, behavioural conduct and overall self-worth [90]. These competencies are understood to reflect the underpinnings of an individual’s self-worth and are intricately related to the latter, depending on the perceived value individuals’ place on each domain [90]. The SPPA scale uses a “structured alternative format”, with each item requiring the individual to first decide on what kind of teenager he or she is most like, and then respond to whether the description is “sort of true” or “really true” (p.4.) [90]. For each item, a score of 4 represents the most satisfactory, and a score of 1 the least satisfactory self-assessment, after negatively worded items are reverse-coded. Domain scores are obtained by calculating the mean of the five items within each subscale. Subscale scores with means closest to 4 are most positive and reflect a high perception of competency in the domain in question.

The SPPA is reportedly a psychometrically robust measure; with acceptable internal consistency scores for each subscale based on Cronbach’s alpha [90]. Comparable internal consistency of the measure has also been established in populations of students with a learning disability (α = 0.89), and those with behavioural disorders (α = 0.85) [91]. Robustness of the factor pattern for students with learning disabilities, and students with behavioural disorders suggests that domain distinctions are meaningful for these sub-groups, and that the instrument is valid enough to be used effectively in special education research [90]. Validity of the measure in an equivalent Australian sample has been previously substantiated by other researchers [92,93]. Discriminant validity of the scholastic competence and the behavioural conduct subscales among secondary school typically developing students, students with learning disability and behavioural disorders has been substantiated previously [94].

#### Social Skills Rating System

The Social Skills Rating System is a multi-rater instrument with a child, parent and teacher version, designed to assist professionals in screening and classifying children suspected of having significant social behaviour problems [51]. In this study, Secondary Student Form of the SSRS (SSRS-SSF) was used to measure how frequently students engaged in 39 social behaviours, categorised into assertion, self-control, cooperation, and empathy domains. Subscales scores were added to compute total social skills scale scores, with higher scores indicating higher frequency of use of social skills. The SSRS is deemed valid to assess social skills in children with and without special needs [51,95]. Prior research suggests that the total social skills scale version of the SSRS-SSF (frequency rating) has adequate internal consistency (α = .83) to permit its independent use in samples of multi-racial American and Australian adolescents with and without disabilities [96]. The SSRS has been used in several studies as a screening tool [97,98] and as a measure to assess treatment outcomes [99,100]. Studies have also found the SSRS to discriminate between the broad categories of students with and without disabilities [101–103].

#### Psychological Sense of School Membership

Student perception of school belongingness was measured using the 18-item Psychological Sense of School Membership (PSSM) scale [55]. Belongingness within this scale is operationalised in terms of the degree to which a student feels accepted and included within the school [55]. The PSSM is deemed to be useful for research and planning interventions both at the level of the individual and the organisation. Items include statements such as: “I feel like a real part of—name of school”; and “People here notice when I’m good at something”. Approximately one-third of the items are phrased in a negative direction in an attempt to avoid the development of a response set bias. A five-point Likert scale is used to collect responses, with choices ranging from 1 (not at all true) to 5 (completely true). A total mean score is calculated by summing the item scores and dividing them by 18, to give a value ranging from 1 to 5; with a higher score indicative of greater belongingness. The PSSM has been tested on middle school and secondary school students in both urban and suburban communities in the United States of America [55]. The PSSM has satisfactory internal consistency (α = .80) [55] and a test–retest reliability index of .78 (4-week interval) [104] and .56 and .60 for boys and girls respectively (12-month interval) [105]. Positive correlations between PSSM scores and school success [106], Grade Point Average (GPA), academic competence and self-efficacy [107] are documented. Higher PSSM scores indicate greater perceived school belongingness. The PSSM has been shown to discriminate between groups of students predicted to be different in terms of their sense of belonging in school [55].

#### Loneliness and Social Dissatisfaction Scale

To obtain an index of students’ feelings of loneliness and dissatisfaction with peer relations, the Loneliness and Social Dissatisfaction Scale (LSDS) [59] was administered. The rating scale is a self-administered questionnaire for students aged 6–18 years. The 16 primary items are comprised of items on feelings of loneliness (e.g., “I’m lonely”), perceptions of peer relationships (e.g., “I don’t have any friends”), perceptions on how relationship provisions are being met (e.g., “There’s nobody I can go to when I need help”), and perceptions of social competence (e.g., “I’m good at working with other children”). Students were asked to indicate the degree to which each statement was a true description of themselves on a five-point scale ranging from 1 (not at all true) to 5 (always true), with reverse ordering for particular items to minimise response set bias [59]. One total score of loneliness and social dissatisfaction was obtained for each student, as well as subscale scores for loneliness and social dissatisfaction [59]. The authors report satisfactory internal consistency reliability, with Cronbach’s α = .79 [59].

The scale is widely used to assess self-perception of loneliness and social dissatisfaction in children both with and without special needs [63,108,109]. Children’s self-report on this form correlates significantly with peer status derived from sociometric measures, and also with the teacher’s report of the child’s social behaviour (Cassidy & Asher, 1992). The LSDS is designed primarily as an outcome measure and has been used to examine changes in loneliness in young people with physical disabilities [110].

#### School Participation Questionnaire

The nature and extent of participation in school activities within the contexts of physical, social and psychological features of the school environment was assessed by the School Participation Questionnaire (SPQ); a measure developed for this study. Items from the National Survey of School Environments [111], the School Microsystems subscale from the Involvement Microsystems Scale [112], and The Curriculum Framework of Western Australia [113] were incorporated into this questionnaire. Students were asked to report whether 14 school activities were *available* at their school. Availability was operationalised as: ‘offered by the school with appropriate adaptations that make it possible for the student to take part’. Students were also asked to rate how often they participated in each of the 14 activities (if available), on a six-point frequency scale. The original version of the School Microsystems subscale has demonstrated moderate internal consistency (α coefficient = .73) [112].

Exploratory factor analysis was undertaken to ensure the validity of the School Participation Questionnaire, prior to its use in the analysis. A minimum factor loading of .45 was set, and the first three factors were obtained from a Principal Component Analysis. The Kaiser-Meyer-Olkin (KMO) measure of sampling adequacy was .79, above the recommended value of .60, and Bartlett’s test of sphericity was significant (χ^2^ = 509.77, *p* < 0.05). The analysis showed that the first factor (Participation in School Related Activities) explained 23.9% of the variance; the second factor (Participation in Community Activities) explained 9.8% of the variance; and the third factor (Participation in Out of School Activities) explained 8.1% of the variance in participation. The three-factor solution was found to account for 41.7% of the variance in participation.

## Data Management and Analysis

Data were analysed using the Statistical package for the Social Sciences (SPSS v.20). Only 0.9–2.5% of data were missing at scale levels. The estimation maximisation (EM) algorithm and Little’s Chi-square statistic identified data to be missing completely at random, with the probability level set at .05 [114,115]. Standard guidelines recommended by tool developers were followed to replace missing values. Where guidelines were not present, missing values were replaced by mean scores. Independent samples *t—*tests confirmed that the profiles of those whose data were missing for various questions were similar to those who responded.

Normality checks for each independent variable were performed, and appropriate transformations undertaken for variables that departed from normality [115]. Linear regression analyses were run to determine whether differences in subgroup mean scores existed [Disability Vs. Typically developing student (TD)]; and if so, to examine the amount of variability in mean score differences. Univariate Discriminant Function Analysis (DFA) was conducted to identify the independent variables that could most accurately distinguish between students with and without disability. Sensitivity, specificity, overall classification accuracy, PPV, NPV, positive likelihood ratio (LR^+^), and negative likelihood ratio (LR^-^) of each model were tabulated. For more information on how to interpret the indices refer to S1 Appendix.

An attempt was made to identify the independent variables that could best predict the presence of disability in the student sample, using multivariate Discriminant Function and logistic regression analyses. Models were developed using a forward stepwise strategy, with the likelihood ratio used to determine the order of entry of variables. The standardised canonical discriminant function coefficients and the unstandardised function coefficients for discriminant analysis and the Wald statistic for logistic regression were used to evaluate the degree to which each of the variables contributed to the discrimination between the two groups. The contribution of the respective variables to the discrimination depended on the magnitude and the direction of the coefficients.

## Results

### Participant demographics and subgroup divisions

Data from 395 students, their parents and class-teachers were collected. The mean age of the student sample was 11.9 years (*SD* = 0.45 years, median = 12 years). Boys comprised 47.3% (*n* = 187) of the sample. Based on the Australian Bureau of Statistics median income categorisation [116], the majority of the sample (58%, *n* = 224) was from mid-range socio-economic status (SES). A total of 17.5% (*n* = 65) of the sample were reported by a parent or primary care-giver to have a disability. The predominant disabilities included cerebral palsy, ADHD, Autism Spectrum Disorder, learning disabilities, and sensory disabilities (i.e., vision and/or hearing loss).

Univariate DFA models were applied to determine the classification ability of each independent variable in differentiating students with disability from their typically developing peers, based on their physical (SF-36) and mental health functioning (overall mental health, perceived competence, coping), social adaptation (social skills, belongingness, loneliness, social satisfaction) and participation profiles.

Simultaneous linear regression models (in the case of continuous independent variables) were fitted to determine differences in *Mean-values* of the sample due to health status (disability only versus typically developing peers); and χ² analyses were undertaken to estimate whether between group differences in each of the subgroups differed beyond chance.

### Child’s physical and overall health (SF-36)

As shown in Table 1, based on parental report of their child’s physical and overall health status, 60% of students with a disability could be accurately classified. The overall health status of a child was a better marker for disability (PPV for disability = 40%) than physical health status (PPV for disability = 25%). The sample’s parent ratings of physical and overall health were not in the LR interval for being considered potentially useful in differentiating students (i.e., < 0.3 for LR^-^ and > 7.0 for LR^+^) [117], based on the presence or absence of a disability.

**Table 1:** Ability of rating of child’s physical and mental health functioning in differentiating disability in a community mainstream school sample.

Measures	IV Scales	Mean (SD)	Mean (SD)	Between group	CC	SN	SP	PPV	NPV	LR^+^	LR^-^
		DG	TD	mean score Δ	(%)	(%)	(%)	(%)	(%)	(%)	(%)
**SF-36**	Physical health of child parental report (SF-36)^a^	1.03 (.32)	0.88 (0.24)	*p* < .001; *R* ^2^ = .07	60.1	63.1	59.4	24.7	88.4	1.55	0.62
	Overall health of child parental report (SF-36)^b^	2.43 (.90)	1.73 (0.71)	*p* < .001; *R* ^2^ = .12	78.6	47.7	85.1	40.3	94.9	3.19	0.61
**Strengths and Difficulties Questionnaire**	Total SDQ mental health functioning score^c^	2.31 (.64)	1.64 (0.75)	*p* < .001; *R* ^2^ = .11	71.3	78.5	69.8	35.4	93.9	2.60	0.31
	Peer problems subscale^d^	1.04 (.74)	0.56 (0.60)	*p* < .001; *R* ^2^ = .09	70.0	61.5	71.8	35.7	89.8	2.18	0.54
	Hyperactivity subscale^e^	1.46 (.65)	0.98 (0.64)	*p* < .001; *R* ^2^ = .08	62.2	69.2	60.7	27.1	86.2	1.76	0.51
	Emotional problems subscale^f^	1.18 (.65)	0.72 (0.65)	*p* < .001; *R* ^2^ = .07	62.5	66.7	61.4	27.0	90.0	1.75	0.53
	Conduct problems subscale^g^	0.64 (.61)	0.39 (0.51)	*p* < .001; *R* ^2^ = .04	58.7	61.5	58.1	23.7	87.7	1.47	0.66
**Adolescent Coping Scale**	Coping: problem solving^h^	67.71 (12.80)	71.30 (11.23)	*p* <.001; *R* ^2^ = .008	56.8	60.0	56.2	22.4	86.9	1.37	0.71
	Coping: Reference to others^i^	54.47 (17.31)	54.32 (15.53)	ns	47.2	55.4	45.5	17.6	82.8	0.67	2.53
	Coping: Non-productive^j^	51.91 (13.31)	48.49 (12.80)	ns	52.0	49.2	52.6	17.9	83.1	1.04	0.97
**Harter’s Scale of Perceived Competence**	Self-worth^k^	3.12 (0.69)	3.33 (0.61)	*p* < .001; *R* ^2^ = .01	55.5	50.8	56.5	19.7	84.4	1.17	0.87
	Academic competence^l^	2.45 (0.65)	2.94 (0.70)	*p* < .001; *R* ^2^ = .07	64.9	64.6	64.9	28	89.7	1.84	0.54
	Athletic competence^m^	2.68 (0.81)	2.90 (0.75)	*p* = .009; *R* ^2^ = .02	60.6	47.7	63.3	21.5	85.2	1.84	0.54
	Physical appearance competence^n^	2.81 (0.63)	2.85 (0.74)	ns	53.1	52.3	53.2	19.1	84.1	1.12	0.90
	Behavioural conduct competence^o^	3.02 (0.75)	3.17 (0.66)	ns	56.8	50.8	58.1	20.4	84.8	1.21	0.85
	Close friendship competence^p^	2.89 (0.80)	3.36 (0.67)	*p* < .001; *R* ^2^ = .07	68.9	60	70.8	30.2	89.3	2.08	0.57
	Social acceptance competence^q^	2.79 (0.74)	3.20 (0.65)	*p* < .001; *R* ^2^ = .05	69.7	56.9	72.4	30.3	88.8	2.06	0.59

Note. IV = Independent variable; DG = disability group (discriminant variable); TD = typically developing; CC = Correct classification; SN = Sensitivity; SP = Specificity; PPV = Positive predictive value; Negative predictive value; LR^+^ = Positive likelihood ratio; LR^-^ = Negative likelihood ratio; ns = not significant

Log transformed score^a^: higher score = poorer physical health of child

Ordinal score^b^: higher score = poorer overall health of child

Log transformed score^c^: higher score = poorer mental health functioning

Log transformed score^d^: higher score = greater peer problems

Log transformed score^e^: higher score = greater hyperactivity

Log transformed total SDQ score^f^: higher score = greater emotional problems

Log transformed total SDQ score^g^: higher score = greater conduct problems

Total adjusted score^h^: higher scores = greater use of problem solving coping strategies

Total adjusted score^i^: greater use referencing to others

Total adjusted score^j^: greater use of non-productive coping strategies

Mean raw score^k^: Higher score = greater self-worth

Mean raw score^l^: greater academic competence

Mean raw score^m^: greater athletic competence

Mean raw score^n^: greater physical competence

Mean raw score^o^: greater behavioural conduct competence

Mean raw score^p^: greater close friendship competence

Mean raw score^q^: greater social acceptance competence.

### Strengths and Difficulties Questionnaire: Total and subscales

The ability of different scores from the SDQ to correctly classify students with a disability from their typically developing counterparts is displayed in Table 1. The sensitivity of the total SDQ score to correctly screen students with disability was 79%; while the PPV or its ability to correctly identify students with a disability from the mainstream population of students was 35%. The PPV of the each SDQ subscale, in predicting mental health problems in children with a disability ranged from 24%–36%. The sample’s mental health scores were not in the LR interval for being considered potentially useful in differentiating students based on the presence or absence of a disability [117]. As shown in Table 1, group differences in mental health functioning scores explained less than 1% of the variability in scores. We also undertook DFA using the 90% totals score dichotomisation scaling system recommended by the instrument developers. However, performances in all the discriminant indices were worse than using the continuous scores; thus the dichotomised results were not reported.

### Adolescent Coping Scale

Univariate DFA suggested that the sensitivity of students’ coping scores in predicting disability status ranged from below to just above chance (49%–60%). Students’ problem solving coping style had better precision (PPV) than other coping styles in determining disability membership (PPV = 22%). The NPV of each coping subscale to correctly identify typically developing students from a mainstream population of students ranged between 83%–87%. The sample’s coping scores were not in the LR interval for being considered potentially useful in differentiating students based on the presence or absence of a disability [117]. As shown in Table 1, although linear regression analyses revealed significant group differences in coping styles between typically developing students and students with a disability; less than 2% of the variability in coping was explained by these scores.

### Self-Perception Profile for Adolescents

Univariate DFA suggested that based on Harter’s self-reporting competence scales, 53–70% of the disability group could be correctly classified (Table 1). At best, the PPV was 30%. The sensitivity of all competence subscales in correctly identifying students with disability was just above chance (50%), apart from the academic competence and close friendship subscales demonstrating sensitivity of 65% and 60% respectively. None of the sample’s scores were in the LR interval for being considered potentially useful in differentiating students based on the presence or absence of a disability [117]. Although linear regression analyses revealed significant group differences in perceived competence between typically developing students and the students with a disability; less than 7% of the variability in competence was explained by these scores.

### Social Skills Rating Scale

The total social skills scores, presented in Table 2, could correctly classify 60% of the disability group. The PPV of the total social skills score to identify students with disability was 23%. The PPV of the other subscales ranged from 19% to 23%. The sample’s social skills were not in the LR interval for being considered potentially useful in differentiating students based on the presence or absence of a disability [117]. As with other independent variables, regression analyses explained less than 3% of the between-group variability in *Mean-values*.

**Table 2:** Ability of social adaptation factors in differentiating disability in a community mainstream school sample.

Measures	IV Scales	Mean (SD) DG	Mean (SD) TD	Between group	CC	SN	SP	PPV	NPV	LR^+^	LR^-^
				mean score Δ	(%)	(%)	(%)	(%)	(%)		
**SSRS (Frequency)**	Total social skills^a^	53.21 (11.08)	56.18 (10.11)	*p* = .011; *R* ^2^ = .02	60.3	55.4	61.4	23.2	86.7	1.43	0.73
	Assertion^b^	11.56 (3.50)	12.93 (3.44)	*p* = .001; *R* ^2^ = .03	57.9	61.5	57.1	23.3	83.4	0.80	1.65
	Empathy^c^	14.71 (3.70	15.43 (3.31)	ns	54.4	53.8	54.5	20.0	84.8	1.18	0.85
	Co-operation^d^	14.52 (3.39)	15.29 (2.98)	*p* = .03; *R* ^2^ = .01	58.4	43.1	61.7	18.9	83.7	1.12	0.92
	Self-control^e^	12.42 (3.42)	12.55 (3.37)	ns	51.2	61.5	49.0	20.3	85.7	1.21	0.78
**PSSM**	School belongingness^f^	3.78 (.82)	3.90 (0.69)	ns	54.0	44.6	56.0	17.7	82.7	1.01	0.99
**LSDS**	Combined score for LSDS^g^	3.42 (.37)	3.26 (0.32)	*p* < .001; *R* ^2^ = .04	63.8	61.5	64.3	26.6	88.8	1.72	0.60
	Loneliness subscale^h^	2.98 (.41)	2.80 (0.34)	*p* < .001; *R* ^2^ = .04	64.6	58.5	65.9	26.6	88.3	1.71	0.63
	Social dissatisfaction subscale^i^	11.35 (3.79)	9.92 (3.66)	*p* = .002; *R* ^2^ = .03	63.8	58.5	64.9	26.0	88.1	1.67	0.64

Note. IV = Independent variable; DG = disability group (discriminant variable); SSRS = Social Skills Rating Scale; PSSM = Psychological Sense of School Membership (scale); LSDS = Loneliness and Social Dissatisfaction Scale; TD = typically developing; CC = Correct classification; SN = Sensitivity; SP = Specificity; PPV = Positive predictive value; Negative predictive value; LR^+^ = Positive likelihood ratio; LR^-^ = Negative likelihood ratio; ns = not significant

Total score^a^: higher scores = more frequent use of total social skills

Total score^b^: greater frequency of assertion behaviours

Total score^c^: greater frequency of empathy behaviours

Total score^d^: greater frequency of cooperation behaviours

Total score^e^: greater frequency of self-control behaviours

Mean raw total score^f^: higher scores = greater belongingness in school

Log transformed total score^g^: higher scores = greater LSDS

Log transformed total subscale score^h^: higher scores = greater loneliness in school

Log transformed total subscale score^i^: higher scores = greater social dissatisfaction in school.

### Psychological Sense of School Membership

Students’ school belongingness scores could correctly classify 54% of the mainstream student sample, using disability as a discriminant factor. The sensitivity of the school belongingness score in correctly screening students with disability based on their belongingness scores was 45% while its specificity (or ability to correctly identify typically developing students based on their belongingness scores) was 56%. The PPV or the ability to correctly identify students with a disability from the mainstream population of students based on their belongingness scores was 17%.

### Loneliness and Social Dissatisfaction Scale

The loneliness and social dissatisfaction scaled-score (LSDS), and its subscales focussing on loneliness only and social dissatisfaction could correctly classify between 64–65% of students with disability. The PPV or the ability of these subscales to identify students with disability with lower loneliness and social dissatisfaction scores from a population of mainstream students was 26%. The sample’s LSDS scores were not in the LR interval for being considered potentially useful in differentiating students based on the presence or absence of a disability [117]. As shown in Table 2, group differences in mean belongingness and loneliness scores explained less than 4% of the variability in scores.

### School Participation Questionnaire

Based on the frequency of student reported participation in school activities, one could accurately identify between 19%–21% (PPV) of students with disability from a mainstream sample of students with and without disabilities (Table 3). The sample’s participation scores were not in the LR intervals for being considered potentially useful in discerning students based on the presence or absence of a disability [117]. Linear regression analyses revealed no significant differences in participation components between typically developing students and a subgroup with a disability.

**Table 3:** Ability of the students’ extra-curricular activity participation profiles to differentiate disability in a community mainstream school sample.

Measure	IV Scales	M (SD) DG	M (SD) TD	Between group Δ M	CC (%)	SN (%)	SP (%)	PPV (%)	NPV (%)	LR^+^	LR^-^
**School Participation Questionnaire**	Frequency of Participation in School Related Activities^a^	4.45 (1.02)	4.69 (0.71)	ns	69.3	27.3	78.3	21.1	83.5	1.25	0.93
	Frequency of Participation in Community Activities^a^	4.20 (0.85)	4.26 (0.88)	ns	50.0	59.1	48.0	19.8	84.3	1.14	0.85
	Frequency of Participation Out of School Activities^a^	2.64 (1.02)	2.64 (1.03)	ns	53.8	47.7	55.1	18.5	83.1	1.06	0.95

Note. IV = Independent variable; DG = disability group (discriminant variable); TD = typically developing; CC = Correct classification; SN = Sensitivity; SP = Specificity; PPV = Positive predictive value; Negative predictive value; LR^+^ = Positive likelihood ratio; LR^-^ = Negative likelihood ratio; ns = not significant

^a^Total score: higher scores = greater participation.

### Summary of results

Using a series of univariate discriminant function analyses and other screening indices, we set out to examine the ability of several measures in predicting the presence of disability in a mainstream sample of students with if a student has a disability. The sensitivity of the included scales ranged from 27.3% to 78.5%, with an overall mean sensitivity of 56.2%, while the specificity of the scales ranged from 45.5% to 85.1%, with an overall mean specificity of 61.2%.

Our results indicated that the ability of the included scales for correctly classifying (CC) disability ranged from 47.2 to 78.6%, with an overall mean CC of 60.4%. The CC indicates the proportion of true results (both true positives and true negatives) in the sample. This means that most scales have nearly an equal chance of either correctly or incorrectly classifying a person as having a disability. However, the sensitivity and specificity of a test cannot be used to estimate the probability of a child having a disability [118]. Given that PPV describes the probability of having a disability when the student has already been classified as having a disability, PPV becomes an important index [119]. The PPV ranged from 17.6% to 40.3%, with an overall mean PPV of 24.2%.

Across the board, the sensitivity of independent variables to correctly identify students with a disability was 31.9% higher than the related PPV. The lower PPV scores suggest that the included independent variables had only a small chance (17.6% to 40.3%) to correctly identify students with a disability from the mainstream population of students. These findings suggest that students with a disability do not differ enough from their typically developing counterparts on each independent variable measured in this study, for them to be identifiable. Thus, based on a univariate DFA of the current sample, the presence of a disability did not seem to impact on their physical and mental health, social adjustment, and participation outcomes.

Given that the instruments included in the study are also used to measure differences between groups, we calculated mean score differences for each of the independent variable scales between the disability and typically developing group. For most independent variable scales (19 out of 29) there were significant differences when comparing the mean scores of the disability group with the typically developing group. The overall trends suggest that, when mean performance on a measure is used to determine if groups differ, the independent variable scales used in the study do well in detecting differences. However, when they are used to differentiate and classify groups they performed poorly.

We calculated the *R* ^*2*^ to determine how much of the variance was explained by the independent variables. The results indicated that the *R* ^*2*^s ranged between 1 and 12%.

The extremely low *R* ^*2*^ values indicate that there are a number of other determinants for each outcome that were not included in the equation, making the models poor predictors for each outcome. However, the models do identify a clear difference in the mean scores between the disability and the typically developing groups (*p <* .05) for some outcomes. Hence the low *R* ^*2*^ explains why the sensitivity scores are low, as their ability to predict the disability status of a particular respondent (having a disability or typically developing) is very poor because of the large variance. There are many other factors that influence the value of the dependent variable other than the disability status.

### Logistic regression analysis

By applying a multivariate DFA with a combination of four independent variables: presence of hyperactivity-inattention (parental report); identification of the child to have problems in peer relationships (parental report); lower perceived academic competence (child self-report); and physical health status (parental report), the groups with and without disability appeared to separate more clearly (λ = 0.816, χ^2^(4) = 79.03, p < .001), with an *R²*-canonical = 0.429, and 83.8% correct classification (sensitivity = 28.8%, specificity = 94.8%, PPV = 52.78, NPV = 86.9). Table 4 shows the standardised canonical coefficients and the structure weights of the independent variables that contributed in this multivariate model.

**Table 4:** Predictors, standardised, and unstandardised coefficients for the discriminant analysis model and logistic regression model.

	Logistic regression							DFA	
Independent variables	*B*	SE	Wald	Sig.	Exp(B)	Lower 95% C.I. for Exp(B)	Upper 95% C.I. for Exp(B)	Standardised Canonical Discriminant Function Coefficients^1^	Structure weights^2^
Overall health of the child^a^	.75	.19	14.84	< .001	2.12	1.44	3.12	.42	.66
SDQ- Hyperactivity	1.06	.45	5.45	.020	2.89	1.18	7.05	.39	.55
SDQ—peer problems^b^	-	-	-	-	-	-	-	.33	.57
SPPA—Close friendship competence	-.53	.20	6.96	.008	.58	.39	.87	-.29	-.54
SPPA—Academic competence	-.47	.22	4.49	.034	.61	.39	.96	-.36	-.53

Note:

^1^The standardised discriminant function coefficients serve the same purpose as beta weights in multiple regressions (partial coefficient): they indicate the relative importance of the independent variable in predicting disability status within the study population. They allow you to compare variables measured on different scales. Coefficients with large absolute values correspond to variables with greater discriminating ability

^2^The structure matrix table shows the correlations of each variable with each discriminant function; the correlations then act similarly to factor loadings in factor analysis

^a^greater score = worse health

^b^SDQ—peer problems was not a significant predictor in the logistic regression model

SPPA = Self—Perception Profile for Adolescents.

The logistic regression analyses suggested that the full model was statistically significant against a constant only model, indicating that the same set of independent variables were significantly associated with the disability status (χ^2^ = 61.534_*(4)*_ < 0.001). However, Nagelkerke’s pseudo *R* ^*2*^ of 0.243 indicated that its predictive value may not be strong. Prediction success overall was 86% (97.9% for the typically developing group and 27.3% for the disability group). The Wald criterion demonstrated that close friendship competence, presence of peer problems, hyperactivity, and poor physical health significantly predicted disability. The Odds Ratios indicated that:

- presence of hyperactivity increased the odds of having a disability by factors of 2.89;
- poor overall health increased the odds of having a disability by 2.13;
- a unit reduction in perceived close friendship competence decreased the odds of having a disability by a factor of 0.59; and
- unit reduction in academic competence decreased the odds of having a disability by 0.62.

We conducted logistic regression to determine which factors were significantly associated with having a disability on a multivariate level. While significant, the multivariate analyses optimising the inclusion of all independent variables suggested that the sensitivity was at best 28.8% to correctly identify a person with a disability. Moreover, the low overall *pseudo R* ^*2*^ of the model raises the issue of its generalisability. Indeed, the only findings that were congruent with previous research, given these multivariate analyses were that poor health and hyperactivity increased the odds of having a disability about two times, while poor close perceived friendship and academic competences predicted disability with roughly the same magnitude. Nevertheless, a summation of all the findings of the current study suggests that schools should not necessarily expect poor academic, social, mental health and participatory outcomes in students with a disability. The analysis shows that children with these conditions obtained scores on these instruments which are not very different from those which typically developing children obtained.

## Discussion

The premise for this paper was to determine if we can identify adolescents with disabilities from a community sample of young adolescents in their final year of primary school with commonly used screening and outcome measures. That is, can we employ commonly used screening and outcome measures to accurately predict if children, known to have a disability (i.e., a predefined group), are accurately classified by the measures as having a disability? The answer to the question—in short—is that the measures used in this study performed poorly in correctly classifying children with a disability.

Over the past several decades, educational policies in many countries have been geared towards the inclusion of students with disabilities in mainstream educational programs [120,121]. Teachers are likely to receive information about their next inbound students and may form assumptions about student functioning based on the knowledge that the student has a disability. However, existing research presents mixed findings on whether students with disabilities studying in mainstream schools differ from their typically developing peers. Notably, these studies have used convenience sampling techniques, focussed on few disability subtypes (mainly mild intellectual disability, learning disability, ADHD, Autism Spectrum Disorders), and used univariate tests to substantiate group differences (e.g., [10,11]). The present study is the first to actually examine whether or not it is possible to accurately determine the disability status of a student based on their physical and mental health, social adjustment, and participation outcomes. The measures we used did not accurately capture the factors that are required to separate them into distinct groups: typically developing students and students with a disability. None of the measures had LR^+^ values that can be considered useful in differentiating students with and without disabilities. This finding further substantiates our claim that in a community sample of students with and without disabilities, the measures used in the current study had limited discriminant ability in accurately awarding membership or identifying students *at-risk* who would be eligible for further assessment.

Measures can have different prognostic and/or analytical functions. Therefore measures can be prognostically used to: a) predict a later outcome; b) determine suitability for a particular intervention; c) report on the responsiveness to a particular intervention; or d) determine the amount of intervention required (dosage) [122]. Measures may also be used analytically to: a) explain or understand the contexts; b) classify or identify subgroups of interest; c) allow exploration of relationship between factors; d) detect within-subject change or between-subgroup differences; e) enable comparison of groups of interest to other population subgroups or norms [122]. If an outcome measure is used to evaluate changes in a person over time, the measure must be able to detect this change [123]. In this study we were specifically interested in the ability of the measures to accurately classify and identify a subgroup (i.e., disability), that is, the identification accuracy of the measures.

Identification accuracy, which refers to an assessment’s ability to accurately diagnose the presence or absence of a condition, is arguably of greater importance than other psychometric criteria, as it indicates the overall precision of making a diagnosis. A discriminant analysis is conducted, which evaluates the measure’s convergent validity to judge its ability to distinguish typical from atypical functioning. Discriminant analysis is carried out using mathematical calculations that contrast different variables, and that take into account variance in scores to reach an overall identification. The sensitivity and specificity data assist researchers and clinicians to gauge the overall identification accuracy of an assessment. Furthermore, if the diagnosis of the condition is known, the PPV should be included as an important index.

Identification accuracy may vary due to the prevalence of a disorder; and the population or setting (clinical vs. community). This means that even if a screening instrument is psychometrically valid and reliable, it may be unlikely to be helpful in identifying individuals *at-risk* if it is not usable within the given setting [124]. Screening is a procedure designed to identify people who have, or who are at risk of having, an illness, disease or disorder [125,126]. Screening is an initial procedure to determine who is eligible for further assessment, and can be used to identify those who are likely to benefit from immediate interventions or preventive counselling because they are considered to be *at-risk*. For example, the utility of the SDQ is different in clinical versus community populations [127,128]. In a clinical population, we assume the presence of psychosocial problems. Therefore, the SDQ should inform us about types of psychosocial problems, the duration, and perception of these problems. In a community setting, we assume the presence of some but not all psychosocial problems; hence, the SDQ should be sensitive in detecting those children in the community who are at risk of having psychosocial problems.

When using group difference indices that report on the magnitude of differences between and within groups (e.g., using *Mean-values*), researchers are able to identify patterns; however, researchers do not tend to report on the classification of the groups. The *R* ^*2*^ is an indicator of how useful the measure is to predict a person’s membership of a group based on their score. In our study the *R* ^*2*^s were very low (1–12%), indicating that the assessments used were poor predictors of students being classified as having a disability. As such, no combination of measures could adequately separate respondents into their correct *disability* category. This is of concern as both the SDQ and SSRS are screening tools that are routinely used to both classify children as being *at-risk* of having a disability, as well as outcome measures to report on effect size following an intervention. Our findings suggests that these measures may be appropriate for use as outcome measures in calculating changes over time in groups (responsiveness), but the groups need to be predefined. However, in an earlier study we found that the SSRS has a large measurement error (ME) [96], a construct closely related to responsiveness. ME (i.e., variability within stable subjects) sets the boundaries of the minimal detectible true change of an outcome measure. Thus, to evaluate true change over time, both the responsiveness and the ME needs to be taken into account where by the responsiveness values need to be wider than the ME. Therefore, the combined findings suggest that the SSRS may not be useful as either screening or an outcome measure. Moreover, the findings highlight the importance for researchers to not only report the differences in *Mean-values* when comparing two or more groups, but also to calculate and report the *R* ^*2*^. *R* ^*2*^ estimates the component of variance in the outcome which is attributable to the set of independent variables in the model.

There is a real need for clinicians and researchers to understand issues related to validity and reliability that accompany the use of measures as part of their diagnostic battery. First and foremost, the stated purpose of the measure intended for use should match the clinical purpose. The purpose of a measure is an important component of any assessment tool, as assessments are conducted for very different diagnostic reasons. For instance, some assessments are administered to diagnose the presence or absence of a condition, as well as to determine the severity level of the condition or to establish the required dosage. As such, researchers and clinicians need to be cognisant of the purpose of a given test in order to collect data reflecting their diagnostic needs. Importantly, some measures might purport to serve a specific purpose, but offer no data to substantiate the validity of using a test for that purpose.

Second, researchers and clinicians must carefully decide which psychometric properties of a given measure should be considered as most essential and, thus, more important to focus upon in selecting assessments for diagnostic use. One of the most important considerations for clinicians in selecting a measure must be the identification accuracy. However, it is likely that information from multiple sources, collected in various environments, is required for making appropriate clinical decisions.

## Conclusion and Future Direction for Research

By using a cross-sectional design and a sample representative of students with and without disabilities, studying within the mainstream school system, the current study concludes that the measures used to determine physical and mental health, social adjustment and participatory outcomes were not helpful in distinguishing between students with and without a disability.

Our findings also highlight the importance of considering the design and purpose of measures. Some measures are designed to serve the function of screening tools and as such to correctly classify and accurately predict grouping; while other measures are designed to accurately measure change over time in a predefined group (responsiveness). Screening measures therefore need to have sound sensitivity, specificity, PPV and NPV and we are less concerned with responsiveness. Conversely, outcome measures that are designed to accurately detect change over time need to be responsive; but the groups under investigation need to have been classified a priori. As such, researchers and clinicians need to familiarise themselves with the relative importance of various psychometric properties in relation to measurement functions and be cautious in matching the function of the measures with their research and clinical needs.

## Supporting Information

S1 Appendix (DOCX)
