# Supplementary material for: Should Schools Expect Poor Physical and Mental Health, Social Adjustment, and Participation Outcomes in Students with Disability?
Source: PLoS One. 2015 May 12;10(5):e0126630. doi: 10.1371/journal.pone.0126630 (PMC4429077; doi:10.1371/journal.pone.0126630)
Supplement: S1 Appendix — (DOCX) [file pone.0126630.s001.docx]

# S1Appendix

Sensitivity and specificity are the most commonly documented indices of screening efficiency. Sensitivity is the proportion of people with disease who will have a positive result [[117](#_ENREF_117)]. The specificity of a test is the proportion of people without the disease who will have a negative result [[117](#_ENREF_117)]. Thus, a test with a test with a high sensitivity is useful for clinicians in ‘ruling out’ a disease if a person tests negative; while a test with a high specificity is useful for ‘ruling in’ a disease if a person tests positive [[129](#_ENREF_129)]. Sensitivity tells us nothing about whether or not some people without the disease would also test positive and, if so, in what proportion; while specificity tells us nothing about whether or not some people with the disease would also have a negative result and, if so, in what proportion.

Sensitivity and specificity however do not take into account false positives (Type 1 error ) or false negatives (Type 11 errors) respectively, and consequently may necessitate the need for further follow-up or not treating those with problems [[130](#_ENREF_130), [131](#_ENREF_131)]. For example, some instruments may have a relatively greater strength to accurately identify all individuals with a disorder (high sensitivity); but at the cost of having a higher rate of false positives (Type 1 error). While, other instruments may have a relatively greater strength to accurately identify all individuals without a disorder (high specificity), yet this ability is likely to come with the cost of having a higher rate of false negatives (Type 11 error; or at greater risk of missing cases or not treat those with problems). A sensitive test will have a few Type 11 errors; and increasing the specificity of the test, lowers the probability of a Type 1 error [[131](#_ENREF_131)]. Because sensitivity and specificity are defined on the basis of people with or without a disease; they do not have practical use when it comes to helping the clinician estimate the probability of disease in individual patients. The accuracy of the test (correct classification; rand accuracy) is the proportion of true results (both True positives and True negatives) in the population [[132](#_ENREF_132)].

Positive and negative likelihood ratios (LR^+^ , LR^-^ ) provides information on how a positive or negative test result changes the likelihood of a person to have a certain diagnosis [[130](#_ENREF_130)]. The Odd’s ratio OR ^D^ summarises the discriminative ability of a test and is computed by dividing the positive and negative likelihood ratios (LR^+^ /LR^-^). The LR^+^, the LR^-^ , and the OR^D^ are interpreted according to the rule of thumb described in Fischer, Bachmann and Jaeschke [[116](#_ENREF_116)], where potentially useful tests (i.e. may alter clinical decisions) usually are characterised by LR^+^ greater than 7 or LR^-^ less than 0.3, or an OR^D^ above 20.

Sensitivity and specificity of a test cannot be used to estimate the probability of a client having a disease [[117](#_ENREF_117)]. Positive and negative predictive values (PPV, NPV) describe the probability of having disease once the results of the test are known [[118](#_ENREF_118)]. PPV and NPV vary with changing prevalence of disease. The higher the prevalence, the higher the PPV, which is the more likely a positive result is able to predict the presence of disease. When the prevalence of disease is low, the PPV will also be low, even when using a test with high sensitivity and specificity.
